# Association of helicobacter pylori infection with serum magnesium in kidney transplant patients

**DOI:** 10.12861/jrip.2014.29

**Published:** 2014-12-01

**Authors:** Massoud Hafizi, Saeed Mardani, Ali Borhani, Ali Ahmadi, Parto Nasri, Hamid Nasri

**Affiliations:** ^1^Department of Infectious Disease, Shahrekord University of Medical Sciences, Shahrekord, Iran; ^2^Department of Internal Medicine, Division of Nephrology, Shahrekord University of Medical Sciences, Shahrekord, Iran; ^3^Department of Epidemiology and Biostatistics, Falculty of Health, Shahrekord University of Medical Sciences, Shahrekord, Iran; ^4^Department of Nephrology, Division of Nephropathology, Isfahan University of Medical Sciences, Isfahan, Iran

**Keywords:** Helicobacter pylori, Magnesium, Chronic renal failure, Kidney transplantation

## Abstract

**Introduction:** Few studies are available regarding the various promoting factors of *H. pylori* infection in kidney disease patients especially renal transplant individuals.

**Objectives:** This study was therefore conducted to examine the association of serum magnesium with *H. pylori* infection among kidney transplant patients. This cross-sectional investigation was conducted on a group of stable kidney transplant patients. Peripheral venous blood samples were collected for biochemical analysis after an overnight fast, Also urea breath test (UBT) was conducted for patients.

**Patients and Methods:** A total of 50 cases was enrolled to the study. Mean serum magnesium value of the patients was 1.98 ± 0.62 mg/dl. Serum magnesium level in positive *H. pylori* patients was more than negative* H. pylori* patients (p=0.0005). In this study population, there was no significant difference in serum intact PTH, calcium, alkaline phosphatase, albumin levels and body mass index (BMI) between males and females or* H. pylori* positive and *H. pylori* negative subjects (p>0.5).

**Conclusion:** It is possible that, magnesium aggravates H. pylori infection in kidney transplant patients through the mechanisms like hemodialysis, which we had reported previously. However, more studies are necessary to prove the association of magnesium with H. pylori infection in renal transplant patients and finding the clinical relevance of our findings.

**Implication for health policy/practice/research/medical education:**

It is possible that, magnesium aggravates H. pylori infection in kidney transplant patients through the mechanisms like hemodialysis, which we had reported previously. However, more studies are necessary to prove the association of magnesium with H. pylori infection in renal transplant patients and finding the clinical relevance of our findings.

## Introduction

Helicobacter pylori (*H. pylori*) infection is detected to be closely associated with upper gastrointestinal disease, like gastro-duodenal ulcers, chronic gastritis, and gastric cancer (1,2). Moreover, renal transplant patients often complain of digestive symptoms. In fact, most renal transplant recipients will have various gastrointestinal complications which are mostly due to opportunistic infections but can also be caused by immunosuppressive drug regimens (2-4). A renal transplant recipient may be affected by mucosa associated lymphoid tissue which is a gastric lymphoma (2-5). This lymphoma may respond to *H. pylori* eradication therapy (3-6). *H. pylori*, infection is a major worldwide health problem, with an estimated global prevalence of approximately 50% too (4-6). *H. pylori* is a spiral-shaped gram negative flagellate bacterium that occupies the mucosal layer of the gastric epithelium (1-3). Diagnosis of *H. pylori* infection can be achieved with both invasive and noninvasive tests. Noninvasive evaluations for the diagnosis of *H. pylori* infection, which are based on analysis of samples of breath, blood, or stool, have been progressed (2-5). Serological tests are unable to discriminate active from pastinfection. Noninvasive tests are valuable for primary diagnosis, when a treatment indication exists, or to observe treatment success or failure. Urease breath test (UBT), could be used both safely and cost effectively to screen *H. pylori-*positive patients and has been shown to be excellent in performing (1-4). The prevalence of *H. pylori* infection is associated the socioeconomic status and living conditions during early life (2,3). However, few studies are available regarding the various promoting factors of *H. pylori* infection in kidney disease patients especially renal transplant individuals. Previously, we had shown the positive association of *H. pylori*–IgG antibody with plasma magnesium in a group of maintenance hemodialysis patients (7).

## Objectives

This study was therefore conducted to examine the association of serum magnesium with *H. pylori* infection among kidney transplant patients.

## Patients and Methods

### Patients

This cross-sectional investigation was conducted on a group of stable kidney transplant patients. Patients were studied between September 20013 to January 2014. The study was carried out in the clinic of Shahrekord University of Medical Sciences of Iran. All patients signed the consent form for participation. All patients were examined and their medical history, including the length of time they received a kidney for transplantation and their treatment protocols were recorded. The basic immunosuppressive regimen of the recipients contained of a combination of cyclosporine at a mean dose of 190 ± 60 mg/d (median: 200 mg/d) and prednisolone 7.5 mg/d and mycophenolate mofetil a dose of 1500 ± 500 mg/d (median: 1500 mg/d). Exclusion criteria included, any active or chronic infection, intake of any antibiotic or non-steroidal anti-inflammatory drugs during the past two months and taking drugs that affect gastric acid production during the past two months, like, antacids, proton pump inhibitors and H2 receptor antagonists and also the presence of acute rejection (8-10). Body mass index (BMI) was calculated by weight in kilograms/height in square meters (11).

### Laboratory methods

Peripheral venous blood samples were collected for biochemical analysis after an overnight fast, including serum creatinine, blood urea nitrogen (BUN), albumin (Alb), serum magnesium (Mg), phosphorus (P), calcium (Ca) and alkaline phosphatase (ALP), using standard kits after an overnight fast. The estimated glomerular filtration rate (eGFR) values were calculated according to the modification of diet in renal diseases (MDRD) formula (12,13). Serum *H. pylori*-specific IgG antibody titer was measured by the enzyme linked immunosorbent assay (ELISA) method using monobind kit (USA) (normal range<20 U/mL). Intact parathormone (iPTH) using Cobas kit, (Roche) was measured by ELISA method (normal range of values is 15 pg/mL to 65 pg/mL). Also urea breath test (UBT) using heliprobe breathcard™/Helikit; (Sweden) was conducted for patients.

### Ethical issues

The study was approved by the institutional ethics committee of Shahrekord University of Medical Sciences. A written informed consent was obtained from all the study participants.

### Statistical analysis

The data was analyzed with Stata 12 (Stata Corporation, College Station, Texas) using descriptive analysis, proportion, mean and standard deviation, confidence interval 95%, Shapiro-Francia W’ test, Student’s t-test, ANOVA, Fisher’s exact and Pearson’s Chi-squared test. For normalization of the data, logarithms of 10 or SE/Robust were used. P values of less than 0.05 was assume d to be significant (p<0.05).

## Results

A total of 50 cases was enrolled to the study. The age of the patients ranged from 28 to 72 with the mean (SD) age of 47.4 ±10.4 years. Demographic and biochemical data of the study patients was shown in  Table 1. 4% of the patients were under 30 years old, 50% between 31-49 years old, and 46% over 50-74 years old. 56% of the patients (n: 28) were male. Mean (SD) of kidney, transplantation in patients was 43.3 ± 20.5 months (range: 8-80). Patients stratified by time after they received a transplanted kidney 8% less than 12 months, 38% between 12-36 months, 42% between 37-72 months and 12% between 73 to 80 months. Comparison sex and duration of kidney transplantation in the study patients was shown in  Table 2. Mean serum magnesium value of the patients was 1.98 ± 0.62 mg/dl and mean of serum creatinine value was 1.36 ± 0.4 mg/dl. In this study, positive significant correlation between serum magnesium and creatinine in total patients was seen (r= 0.32, p= 0.02). Positive significant correlation between serum magnesium and duration of kidney transplantation in positive *H. pylori* patients was detected too (r= 0.82, p= 0.0001). According to UBT and IgG tests, the prevalence of positive *H. pylori* in patients was 38 % and 34% respectively. Mean (SD) serum magnesium of the patients without *H. pylori* was 1.75 ± 0.65 mg/dl and this value in patients positive *H. pylori* was 2.35 ± 0.3 mg/dl. Serum magnesium level in positive *H. pylori* patients was more than negative *H. pylori* patients (p=0.0005). The mean of creatinine in negative and positive *H. pylori* patients was 1.72 ± 0.3 and 1.15 ± 0.2 respectively. Serum creatinine in positive *H. pylori patients* was more than negative *H. pylori* patients (p=0.001). Mean (SD) of biochemical factors was shown in  Table 3. In this study population, there was no significant difference in serum intact PTH, calcium, alkaline phosphatase, albumin levels and eGFR, and also BMI between males and females or *H. pylori* positive and *H. pylori* negative subjects (p>0.5). Correlation coefficients between serum magnesium and others parameters were shown in  Table 4.

**Table 1:** Demographic and biochemical data of the study patients

**Parameter**	**Mean ± SD**	**Median**	**Minimum**	**Maximum**
Age (years)	47.4 ± 10.4	47	28	72
Serum creatinine (mg/dl)	1.36 ± 0.4	1.3	0.5	2.2
Serum magnesium (mg/dl)	1.98 ± 0.62	2.05	0.09	2.7
Intact PTH (pg/mL)	25.1 ± 45	15.5	1.2	317.3
Serum phosphorus (mg/dl)	4.19 ± 0.63	4.1	2.7	5.9
eGFR (cc/min)	76.75 ± 26	74.5	35.4	169.8
Serum calcium (mg/dl)	9.19 ± 0.43	9	8.4	10.2
Serum alkaline phosphatase (IU/ml)	203.5 ± 66	186	91	351
Serum albumin (g/dl)	3.88 ± 0.25	3.9	3.4	4.3
BMI (kg/m^2)^	23.6 ± 3.4	23.7	16.04	30.4

**Table 2:** Frequency of gender and duration of kidney transplant in the study patients

**Parameter**		**Total**	***H. pylori*** ** negative**	***H. pylori*** ** positive**	**P-value**
Gender	Male	28 (56)	11 (39.2)	17 (60.8)	0.534
	Female	22 (44)	8 (36.4)	14 (63.6)	
Duration of kidney transplantation (month)	<11	4 (8)	4 (12.9)	0	0.032*
	12-36	19 (38)	14 (45.1)	5 (26.3)	
	37-72	21 (42)	12 (38.7)	9 (47.3)	
	73-80	6 (12)	1 (3.2)	5 (26.3)	

*P values of less than 0.05 were assumed to be significant (p<0.05).

**Table 3:** Demographic and biochemical data of the study patients with *H. pylori* and without *H. pylori* infection

**Parameter**	***H. pylori*** ** negative**	***H. pylori*** ** positive**	**P-value**
Age (years)	48.2 ± 9.7	46.15 ± 11.6	0.502
Serum creatinine (mg/dl)	1.15 ± 0.2	1.72 ± 0.3	0.0001*
Serum magnesium (mg/dl)	1.75 ± 0.6	2.35 ± 0.3	0.0005*
Intact PTH (pg/mL)	28.3 ± 56	19.9 ± 14.9	0.528
Serum phosphorus (mg/dl)	4.2 ± 0.65	4.12 ± 0.61	0.560
eGFR (cc/min)	76.94 ± 28	76.4 ± 23.4	0.948
Serum calcium (mg/dl)	9.16 ± 0.43	9.23 ± 0.44	0.574
Serum alkaline phosphatase (IU/ml)	205 ± 76	201 ± 50	0.84
Serum Albumin (g/dl)	3.85 ± 0.26	3.93 ± 0.26	0.314
BMI (Kg/m^2^ )	23.5 ± 3.8	23.68 ± 2.9	0.925

* P values of less than 0.05 were assumed to be significant (p<0.05).

**Table 4:** Correlation between serum magnesium and various demographic factors

Variable	***H. pylori negative***		***H. pylori positive***		Total	
	r	P-value	r	P-value	r	P-value
Age (years)	-0.037	0.83	0.27	0.281	0.005	0.96
BMI (Kg/m^2^ )	- 0.14	0.433	0.288	0.261	0.01	0.91
eGFR (cc/min)	0.26	0.138	- 0.22	0.38	0.11	0.42
Duration of transplantation (months)	0.361	0.038*	0.827	0.0001*	0.558	0.001*

* P values of less than 0.05 were assumed to be significant (p<0.05).

## Discussion

In the present study, we found that, the value of serum magnesium level in positive *H. pylori* patients was more than negative *H. pylori* patients. To understand, the aggravating factors of *H. pylori* infection in chronic kidney disease, especially in hemodialysis patients, we previously conducted a study on 44 hemodialysis patients. At this investigation, we detected a significant positive correlation of *H. pylori* antibody with serum magnesium (7). The results of this study suggested the association of serum magnesium with the *H. pylori* infection (7). We also conducted another study on 94 type II diabetic patients who had a mean creatinine clearance value of 62 ± 23 cc/min. In this investigation, there was no significant correlation of serum anti-*H. pylori* specific antibody titer with serum magnesium level (14). It is evident that magnesium ion acquisition is necessary for *H. pylori* (7,15). We assumed that, the elevated serum magnesium level in hemodialysis patients and its higher concentration in the gastric mucosa might improve the gastric colonization of *H. pylori* in patients on hemodialysis, but not in diabetic patients with various stages of kidney failure whom were not undergone hemodialysis yet (7,14-16). Gastric complications are common in patients who under hemodialysis or harbored renal transplantation and *H. pylori* infection is assumed to have an important role in gastrointestinal disease in these patients (1-5,17), on the other hand magnesium is principally  excreted by kidney and magnesium metabolism is disturbed in patients with chronic renal failure (18-23). In fact, elevated serum magnesium level can be a problem in patients on maintenance hemodialysis. While, the kidneys are the major route of excretion of magnesium from the body, increased serum magnesium would be expected in patients with renal failure and in hemodialysis patients (20-27).

## Conclusion

It is possible that, magnesium aggravates* H. pylori* infection in kidney transplant patients through the mechanisms like hemodialysis. However, more studies are necessary to prove the association of magnesium with *H. pylori* infection in renal transplant recipients and finding the clinical relevance of our observations.

## Authors’ contributions

AB, MH and SM conducted the research and gathered the patients. AH analyzed the data. AB, PN and HN prepared the manuscript. All authors read, revised, and approved the final manuscript.

## Conflict of interests

The authors declared no competing interests.

## Ethical considerations

Ethical issues (including plagiarism, data fabrication, double publication) have been completely observed by the authors.

## Funding/Support

This study was extracted from M.D thesis. This study was conducted by a grant from Shahrekord University of Medical Sciences (Grant # 1029).
